# Lexical ambiguity resolution during sentence processing in Parkinson’s disease: An event-related potential study

**DOI:** 10.1371/journal.pone.0176281

**Published:** 2017-05-05

**Authors:** Anthony J. Angwin, Nadeeka N. W. Dissanayaka, Katie L. McMahon, Peter A. Silburn, David A. Copland

**Affiliations:** 1 School of Health and Rehabilitation Sciences, University of Queensland, Brisbane, Queensland, Australia; 2 University of Queensland Centre for Clinical Research, Brisbane, Queensland, Australia; 3 Neurology Research Centre, Royal Brisbane & Women’s Hospital, Brisbane, Queensland, Australia; 4 School of Psychology, University of Queensland, Brisbane, Queensland, Australia; 5 Centre for Advanced Imaging, University of Queensland, Brisbane, Queensland, Australia; 6 Queensland Brain Institute, University of Queensland, Brisbane, Queensland, Australia; University of Akron, UNITED STATES

## Abstract

Event-related potentials (ERPs) were recorded to investigate lexical ambiguity resolution during sentence processing in 16 people with Parkinson’s disease (PD) and 16 healthy controls. Sentences were presented word-by-word on computer screen, and participants were required to decide if a subsequent target word was related to the meaning of the sentence. The task consisted of related, unrelated and ambiguous trials. For the ambiguous trials, the sentence ended with an ambiguous word and the target was related to one of the meanings of that word, but not the one captured by the sentence context (e.g., ‘He dug with the spade’, Target ‘ACE’). Both groups demonstrated slower reaction times and lower accuracy for the ambiguous condition relative to the unrelated condition, however accuracy was impacted by the ambiguous condition to a larger extent in the PD group. These results suggested that PD patients experience increased difficulties with contextual ambiguity resolution. The ERP results did not reflect increased ambiguity resolution difficulties in PD, as a similar N400 effect was evident for the unrelated and ambiguous condition in both groups. However, the magnitude of the N400 for these conditions was correlated with a measure of inhibition in the PD group, but not the control group. The ERP results suggest that semantic processing may be more compromised in PD patients with increased response inhibition deficits.

## Introduction

Language processing impairments in Parkinson’s disease (PD) include deficits to semantic activation [1–3] and sentence comprehension [4,5]. Impairments in processing lexically ambiguous words, that is, words that have more than one meaning (e.g., bank), have also been well documented in PD [6,7]. These problems include selecting the appropriate meaning of an ambiguity presented in a sentence context. For instance, correct interpretation of an ambiguous word like ‘bank’ is made easier when it is placed within a context biasing one particular interpretation (e.g., ‘He placed his money in the bank.’ versus ‘He swam to the edge of the bank.’). However, difficulties utilising such contextual information during ambiguous word processing are evident in PD. Copland et al. [8] utilised a cross modal priming task whereby a spoken sentence biased towards the dominant or subordinate meaning of a sentence-final lexical ambiguity was followed by a visual target word. At a long ISI, control participants displayed priming only for contextually appropriate meanings, indicating that inappropriate meanings had been suppressed. In PD, however, priming for both dominant and subordinate meanings were observed following dominant biased sentences, and no priming was observed following subordinate biased sentences. These results were consistent with a deficit to context-based meaning selection and attentional engagement of the semantic network.

Similar problems in context-based meaning selection were observed in PD patients when presented with lexical ambiguities in a paragraph [9]. More broadly, such difficulties appear consistent with PD related deficits in selecting and suppressing competing motor representations [10], and with evidence to suggest that controlled lexical-semantic impairments in PD may relate to deficits in the inhibition of semantic representations [11–13]. Neuroimaging studies can provide significant insight into the neural mechanisms that underpin ambiguity processing. In a study of healthy adults using functional magnetic resonance imaging (fMRI), Ketteler et al. [14] found significant activation of subcortical circuitry during ambiguity processing, including the thalamus and caudate nucleus. In a later fMRI study of patients with PD, Ketteler et al. [7] found that difficulties with meaning selection during an ambiguity processing task were associated with reduced activity in the left caudate for these patients.

The analysis of event-related potentials (ERPs), which provide excellent temporal resolution, can also be used to explore the neural mechanisms that contribute to language processing. The N400, an ERP component with a negative peak approximately 400ms post stimulus, has proven sensitive to semantic processing [15]. To date, studies of the N400 in PD have been limited. Kutas et al. [16] measured ERPs during a semantic judgement task involving spoken phrases that defined either a categorical or antonymic relationship, followed by a congruent or incongruent target word. The peak amplitude of the N400 was larger in PD than controls for both the antonymic and categorical conditions, whilst measures of mean amplitude showed a larger N400 in PD for the antonymic condition only. Kutas et al. suggested the results could be explained by reduced inhibition of irrelevant semantic information, greater activation of the target or increased reliance on external cues in PD. In contrast, Angwin et al. [17] examined semantic processing in PD using a paired-word semantic judgement task and observed no difference in the N400 mean amplitude for PD patients and controls, however the onset latency of the N400 was slower in PD. Friederici et al. [18] measured ERPs during a passive sentence processing task in PD. Although the absence of a P600 effect for syntactic violations suggested a deficit to controlled syntactic integration processes in PD, an N400 was evident for both PD patients and controls in response to the semantic violations, suggesting that semantic processes involved in the detection of such anomalies were intact.

To date, ERPs have not been used to explore the processing of lexical ambiguities in PD, however numerous studies in healthy adults have demonstrated the utility of the N400 component for investigating ambiguous word processing [19–23]. In a study of healthy adults, Swaab et al. [22] presented sentences biased towards the meaning of sentence final ambiguous words followed by a target related to the contextually appropriate meaning (concordant condition) or the contextually inappropriate meaning (discordant condition). An additional unrelated condition was also included where sentences ended with an unambiguous word that was unrelated to the target. When a short 100ms ISI between sentence and target was used, Swaab et al. observed a smaller N400 effect for the discordant condition relative to the unrelated condition, indicating that activation of the inappropriate meaning was evident. At a longer 1250ms ISI, when the effects of sentence context would be expected to impact meaning selection, different results were obtained. When sentences were biased towards the dominant meaning, the N400 for the discordant condition was similar in magnitude to the N400 for the unrelated condition, indicating that the contextually inappropriate subordinate meaning had decayed or been suppressed. The results of other ERP studies have also been consistent with selective activation of contextually appropriate meanings at a long ISI in healthy young [23] and older adults [24].

Taking such findings into account, the present study aimed to investigate the processing of lexical ambiguities during sentence processing in PD using Gernsbacher et al.’s [25] context verification paradigm. In this task, participants read a sentence and judge whether a following target word is related to the meaning of the sentence. In some trials, the sentence-final word is unambiguous and the target is unrelated (e.g., He dug with the shovel; Target = ACE). For other trials, the sentence-final word is ambiguous and followed by a target related to the contextually inappropriate meaning (e.g., He dug with the spade; Target = ACE). Although both meanings of an ambiguity should initially be active, this activation should be subsequently constrained to the contextually appropriate meaning.

Gernsbacher et al. [25] found that the ability to suppress contextually irrelevant information differed in those with higher versus lower general comprehension skill. In those with higher comprehension skill, reaction times (RTs) to reject test words for the ambiguous condition were slower than RTs to reject test words for the unrelated condition at a short ISI, but this was no longer evident at a long ISI suggesting that suppression of the irrelevant meaning was occurring over time. In contrast, those with lower comprehension skill demonstrated slower RTs for the ambiguous condition at both ISIs, suggesting difficulties with the suppression of irrelevant information. Using the same stimuli, Faust et al. [26] found that healthy older adults demonstrated slower RTs for the ambiguous condition relative to the unrelated condition at both a short and a long ISI. Faust et al. also found that healthy older adults responded less accurately to trials in the ambiguous relative to the unrelated condition at a short ISI, but showed similar accuracy for both conditions at a long ISI. Based on the accuracy results, the authors suggested that the contextually inappropriate meaning was becoming less activated over time. In light of these findings, a long ISI will be utilised in the current study together with the measurement of ERPs to explore the impact of sentence context on ambiguity resolution in PD.

It is hypothesised that control participants will demonstrate similar accuracy and a similar N400 for both the unrelated and ambiguous condition, indicating that the contextually inappropriate meanings have been suppressed. In contrast, it is hypothesised that PD patients will display lower accuracy for the ambiguous relative to the unrelated condition. Further, it is predicted that the PD group will show an N400 for the unrelated condition, but the N400 will be smaller or absent for the ambiguous condition due to an impaired capacity to utilise the sentence context to selectively activate the appropriate word meaning. The relationship between the N400 and performance on a Stroop task was also examined, given the possible influence of executive deficits on language processing in PD [5,27] and the importance of suppression mechanisms for the experimental task [25].

## Methods

### Participants

Twenty PD patients diagnosed according to the UK Brain Bank criteria [28] and with no diagnosis of dementia participated in the study. Due to excessive noise and artefacts in the EEG recordings, data for 4 of these participants could not be analysed and they were subsequently excluded. The remaining 16 participants (13 male; age range 48–82) were compared to a control group comprised of sixteen healthy adults (9 male; age range 54–83). There was no significant difference between the groups in terms of age or education. All PD participants had completed the Parkinson’s Disease Cognitive Rating Scale [29] prior to the current study (within an average of 4.81 months). Participant demographics and other clinical features of the PD group, including the levodopa equivalent daily dosage [30], are presented in Table 1. All participants reported as right-handed, and had no history of any other neurological condition or surgery, drug or alcohol abuse, and were not taking any anti-depressive medications. The study was approved by the Medical Research Ethics Committee at the University of Queensland. All participants provided written informed consent prior to participation.

**Table 1:** Participant demographics and clinical features.

	Control	PD
Age (years)	68.69 (6.86)	67.25 (8.78)
Education (years)	15.50 (3.81)	13.56 (3.60)
Disease duration (years)	n/a	5.19 (2.97)
PDCRS Score	n/a	95.13 (12.44)
Hoehn & Yahr	n/a	2.00 (0.52)
LEDD (mg)	n/a	418.59 (322.76)

PDCRS, Parkinson’s disease Cognitive Rating Scale; LEDD, levodopa equivalent daily dosage. Standard deviations presented in brackets.

### Cognitive testing

Prior to the commencement of testing, all participants completed the Hopkins Verbal Learning Test [31] (HVLT), semantic (animals) and letter (FAS) verbal fluency, and a computerised colour-word Stroop task. The Stroop task was programmed using e-prime 2.0 and consisted of 108 total trials, including 36 neutral trials (the stimulus was a row of X’s), 36 congruent trials (the stimulus was a written word that matched the colour of the font used) and 36 incongruent trials (the stimulus was a written word that differed to the colour of the font used). Participants named the colour of the font for each trial as quickly as possible. Their reaction times were recorded via a microphone plugged into a PST response box and their accuracy was recorded by the experimenter. The order of trials was pseudorandomised and then held constant for each participant.

### ERP task

#### Stimuli

Consistent with Gernsbacher et al. [25], the experimental stimuli consisted of 240 sentences across 3 different conditions; an ambiguous condition, an unrelated condition and a related condition. The stimuli were taken predominantly from Gernsbacher et al., but due to conflicts with Australian spelling and other regional differences, a small number of their original stimuli were modified or replaced with new stimuli.

The ambiguous condition consisted of 80 sentences, 3 to 6 words in length, which ended with an ambiguous word (e.g., He dug with the spade). Each ambiguous final word held at least two meanings according to homograph norms [32–35], and the stimuli were not unequibiased according to the parameters of Simpson and Burgess [36] that biased ambiguities have a dominant meaning that is provided >80% of the time. The target word for each sentence represented the meaning of the ambiguity not captured by the sentence (e.g., the target word ACE is not related to the meaning of the sentence “He dug with the spade”).

For each of the 80 sentences in the ambiguous condition, there was a matching sentence that ended with a semantically comparable unambiguous word (e.g., He dug with the shovel). These sentences formed the unrelated condition. The same target words used for each ambiguous trial were also used for the matching unrelated trial (e.g., The target word ACE is not related to the meaning of the sentence “He dug with the shovel”).

The related condition consisted of 80 sentences similar in length and structure to the ambiguous/unrelated conditions. Approximately half of the sentences in the related condition ended in an ambiguous word (e.g., “She liked the rose”), with the remainder ending in an unambiguous word (“She liked the lake”). For each sentence, a target word was chosen that was related to the meaning of the sentence (e.g., “She had to hide”, Target = conceal; “She liked the lake”, Target = pond). Target words for the related condition differed to the target words used in the unrelated/ambiguous condition.

Two stimulus lists were then constructed. Each list consisted of 80 related trials, 40 unrelated trials and 40 ambiguous trials. The same 80 related trials were included within each list. In contrast, the unrelated and ambiguous trials were counterbalanced across lists, such that if list 1 contained the sentence “He dug with the shovel” (unrelated condition), then the matching sentence “He dug with the spade” (ambiguous condition), would be presented in list 2. In this manner, no target words were repeated within either list. The presentation of lists was counterbalanced across participants.

#### Procedure

Participants were informed that a sentence would appear word-by-word in the centre of the computer screen, followed by a target word. They were asked to decide whether the target word was related to the meaning of the sentence as quickly and as accurately as possible by pressing a ‘yes’ button with their index finger or a ‘no’ button with their middle finger. The responses were recorded using a Psychology Software Tools Serial Response Box.

All stimuli were presented in Arial, 18 point font. At the beginning of each trial, the words “Get ready” were presented for 1500ms, followed by a fixation point ‘+’ for 500ms. The trial sentence was then presented word by word in the centre of the computer screen in lower case letters. Each word in the sentence appeared for 400ms, with a 150ms blank screen preceding each word. After the final word of the sentence there was a blank screen for 850ms followed by the target word, which was presented in uppercase letters and flanked by asterisks (e.g., ** ROSE **). The target word remained on the screen for 3000ms or until the participant responded. The next trial was then initiated automatically after 1500ms. Stimuli were presented over 4 blocks of 40 trials and participants were provided with a short rest break after each block. Prior to completing the experiment, participants also completed a short practice task.

#### ERP recording and analysis

A 128 channel EEG system (Electrical Geodesics, Inc.) was used to record the ERP data. The sampling rate was 500Hz and electrode impedances were maintained below 50kΩ, which is acceptable with the use of high impedance amplifiers [37]. Offline data processing was conducted using Netstation 4.5.1 (Electrical Geodesics, Inc.) and only correctly answered trials were included in the analysis. The data was digitally filtered from 0.1–30 Hz and segmented into 1100ms epochs commencing 100ms prior to the onset of the target word. An ocular artefact reduction procedure [38] was applied to trials that were contaminated by eye movements and blinks, and then any subsequent trials that still consisted of ocular artefacts or that consisted of more than 20 bad channels (defined as reaching amplitudes greater than 200 mV) were excluded from analysis. Any remaining bad channel data was replaced with data interpolated from the remaining channels. The data was re-referenced to the average of all electrodes, with baseline correction performed using the 100ms pre-target baseline.

Three regions of interest in each hemisphere were selected for analysis, with each region consisting of a cluster of four electrodes around the F3/4, C3/4 and P3/4 positions (left frontal– 24(F3), 19, 23, 27; right frontal—124(F4), 4, 3, 123; left central—36(C3), 30, 29, 35; right central—104(C4), 105, 111, 110; left parietal—52(P3), 53, 47, 51; right parietal—92(P4), 86, 98, 97). A time window of 300-500ms was selected for analysis of the N400. The Greenhouse-Geisser correction was utilized for the reporting of all p values whenever violations of sphericity were evident (uncorrected degrees of freedom are reported).

## Results

### Cognitive tests

Table 2 presents the results of the cognitive tests. The semantic and letter fluency data was unavailable for one control participant. The PD group performed more poorly than the control group on the HVLT delayed recall and retention subtests. No group differences were evident for any of the other tasks or conditions.

**Table 2:** Cognitive test scores for the control and PD group.

	Control	PD	p value*
HVLT total recall	24.50 (5.07)	21.75 (5.05)	.135
HVLT delayed recall	9.00 (2.71)	7.25 (2.02)	**.047**
HVLT retention	91.44 (14.70)	79.83 (16.35)	**.043**
Animal fluency	20.13 (4.90)	17.69 (4.18)	.145
Letter fluency (Total FAS)	40.25 (16.55)	36.81 (6.99)	.111
Stroop % accuracy			
Congruent	100	100	n/a
Neutral	99.83 (0.70)	99.48 (1.51)	.410
Incongruent	96.70 (4.33)	90.62 (11.29)	.059
Stroop RT difference			
Incongruent-Neutral	227 (130)	185 (101)	.316
Incongruent-Congruent	238 (118)	197 (97)	.289

HVLT, Hopkins Verbal Learning Test. Standard deviations presented in brackets. Animal and letter fluency data was unavailable for one control participant.

*p values for between group comparisons calculated using independent samples t-tests.

### Ambiguity task

Due to a coding error, one trial from the unrelated condition and one trial from the ambiguous condition was unavailable for approximately half the participants.

#### Behavioural results

Table 3 shows the accuracy and RT data for the unrelated and ambiguous condition for each group. This accuracy data was analysed using a repeated measures ANOVA, with group (PD, control) and condition (unrelated, ambiguous) as independent variables. The analysis revealed no main effect of group, however there was a main effect of condition *F*(1,30) = 24.55, *p* < .001, η_p_^2^ = .450, with subsequent t-tests confirming lower accuracy in the ambiguous condition relative to the unrelated condition for both controls (*p* = .043) and PD (*p* < .001). An interaction effect of group x condition was also evident, *F*(1,30) = 7.65, *p* = .01, η_p_^2^ = .203. In order to explore this interaction further, a measure of interference was calculated (unrelated accuracy minus ambiguous accuracy) and a t-test confirmed that there was significantly larger interference in the PD group relative to the control group (*p* = .011). Although items in the related condition served only as filler trials, a between group comparison of accuracy for the related condition using a t-test revealed similar accuracy for the control (M = 91.56%; SD = 8.34) and PD group (M = 94.30%; SD = 3.23) (*p* = .236).

**Table 3:** Accuracy (%) and reaction time (in ms) for the unrelated and ambiguous conditions of the ERP task for each group.

	Control	PD
Accuracy		
Unrelated	95.91 (7.95)	95.24 (5.50)
Ambiguous	93.21 (8.13)	85.73 (11.80)
Reaction time		
Unrelated	946 (224)	1231 (345)
Ambiguous	1044 (231)	1306 (349)

Standard deviations in brackets.

The RT data for the unrelated and ambiguous conditions was also analysed using a 33repeated measures ANOVA, with group and condition as independent variables. Only correctly answered trials were included in the calculation of RT. Analysis of this data revealed a significant main effect of condition, *F*(1,30) = 47.26, *p* < .001, η_p_^2^ = .612, with subsequent t-tests confirming an interference effect for both groups as evidenced by slower RTs for the ambiguous relative to the unrelated condition in controls (*p* < .001) and PD (*p* = .005) (Table 3). A main effect of group was also evident, *F*(1,30) = 7.05, *p* = .013, η_p_^2^ = .190, indicating slower overall RTs for the PD group. The group X condition interaction was not significant (*p* = .371).

Although ambiguous stimuli were not unequibiased as defined by Simpson and Burgess [36], further analysis of the accuracy and RT data for the ambiguous condition was undertaken to ensure that meaning frequency was not impacting the results. For each participant, accuracy and RT data was recalculated separately for trials where the sentence was biased towards the more frequent meaning (23 trials in list 1; 21 trials in list 2) versus those sentences that biased the less frequent meaning (17 trials in list 1; 19 trials in list 2). ANOVAs were used to analyse this data with group and meaning frequency as independent variables. The analysis of the accuracy data revealed no significant main effect for meaning frequency (*p* = .454) and no group x meaning frequency (*p* = .873) interaction. Analysis of the RT data revealed a significant main effect of group (*p* = .018) due to slower RTs in the PD group, but there was no significant main effect for meaning frequency (*p* = .631) and no significant group x meaning frequency (*p* = .778) interaction. These analyses confirmed that meaning frequency was not impacting responses to the ambiguous condition for either group.

#### ERP results

All incorrect responses were removed prior to ERP analysis. This resulted in the removal of 6.96% of the control group’s data and 7.59% of the PD group’s data. The removal of ERP trials contaminated by artefacts resulted in the additional exclusion of 5.74% of the control group’s data and 10.67% of the PD group’s data from analysis. Overall, there was an average of more than 30 trials available for the analysis of each condition for both the control group (69.44 Related trials; 35.94 Unrelated trials; 33.75 Ambiguous trials) and the PD group (67.31 Related trials; 33.44 Unrelated trials; 30.44 Ambiguous trials).

**Fig 1:**
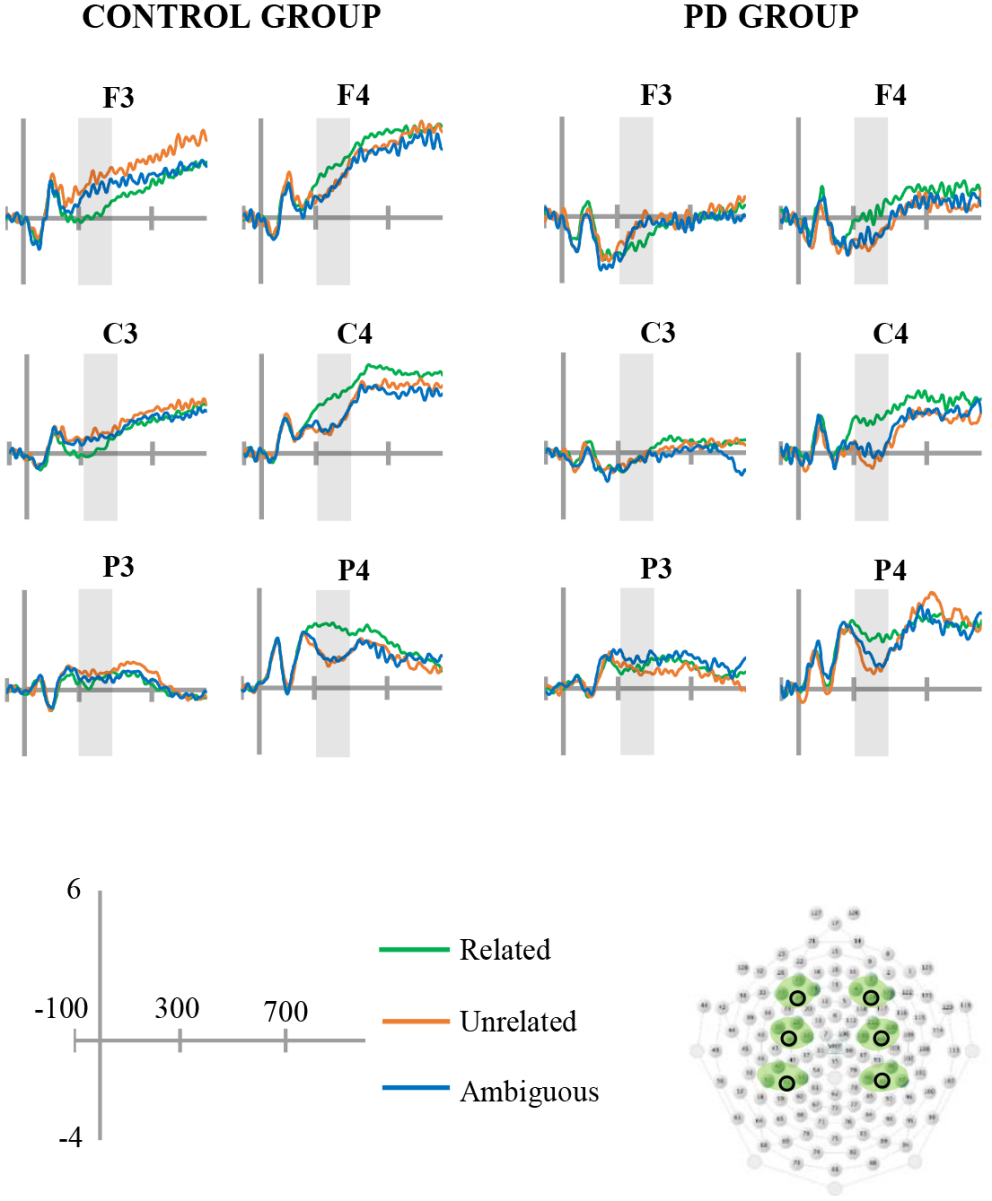
Grand averaged ERP waveforms. Grand averaged ERPs for each group and condition. Electrodes corresponding to positions F3/4, C3/4 and P3/4 are depicted (see black circles in the electrode montage). The N400 time window (300-500ms) is shaded in each waveform and negative is plotted down.

A repeated-measures ANOVA was used to analyse the mean amplitude of the N400 (averaged across the four electrodes within each region of interest) using group (PD, control), condition (Related, Unrelated, Ambiguous), hemisphere (Left, Right) and region (frontal, central, parietal) as the independent variables. Main effects of group, hemisphere and region are not reported. The analysis revealed a main effect of condition *F*(2,60) = 3.81, *p* = .029, η_p_^2^ = .113. Pairwise comparisons between conditions (collapsed across group, hemisphere and region) demonstrated an N400 effect for both the unrelated and ambiguous conditions relative to the related condition (*p* = .014 and *p* = .044 respectively). There was no difference in mean amplitude between the unrelated and ambiguous conditions (*p* = .770). Interaction effects of group x region *F*(2,60) = 7.11, *p* = .007, η_p_^2^ = .191, condition x hemisphere *F*(2,60) = 66.10, *p* < .001, η_p_^2^ = .688, and condition x region *F*(4,120) = 6.01, *p* = .001, η_p_^2^ = .167 were also evident. In order to explore the condition x hemisphere interaction, difference waves were calculated for the unrelated and ambiguous conditions (relative to the related condition), and the magnitude of these difference waves (collapsed across group) was compared between the left and right hemisphere within each region. The analyses confirmed that the N400 effect for both the unrelated and ambiguous condition was more prominent in the right hemisphere of each region (*p* < .001 for all comparisons) (Fig 1). The absence of any interaction involving both condition and group in the ANOVA confirmed that the N400 was similar for both groups.

In order to explore whether the exclusion of errors from the ERP analysis could potentially mask group differences in the N400, the ERP data in the right hemisphere was reanalysed without excluding errors from the calculation of mean amplitude. A repeated measures ANOVA was conducted with group, condition and region as independent variables. The analysis revealed a significant main effect of condition *F*(2,60) = 29.45, *p* < .001, η_p_^2^ = .495, with pairwise comparisons (collapsed across group and region) confirming the presence of an N400 for the unrelated (*p* < .001) and ambiguous (*p* < .001) conditions relative to the related condition, and no difference in amplitude between the unrelated and ambiguous condition (*p* = .088). A group x region *F*(2,60) = 6.60, *p* = .010, η_p_^2^ = .180 interaction was evident, however the absence of any interactions involving both group and condition confirmed that a similar N400 effect was evident for both groups, and that the exclusion of errors was not masking group differences in the N400.

Correlations were then conducted between the N400 difference waves (errors excluded) for the unrelated and ambiguous condition and a measure of inhibition from the Stroop task (calculated by subtracting the mean reaction time of the neutral condition from the mean reaction time of the incongruent condition). These correlations were restricted to the difference waves in the right central and parietal region since visual inspection confirmed that the N400 was most prominent in these regions and because the scalp distribution for the N400 is recognised as typically occurring in the central-parietal region when using word stimuli [39]. The analyses revealed no significant correlations for the control group. In contrast, for the PD group, there was a significant positive correlation between the Stroop inhibition score and the unrelated N400 in the right central region (R = .588, *p* = .017), while the same correlation in the right parietal region fell just outside significance (*p* = .061). There was also a significant positive correlation between the Stroop inhibition score and the ambiguous N400 at the right central and right parietal regions (R = .518, *p* = .040, and R = .524, *p* = .037, respectively).

Finally, the onset latency of the N400 difference waves for the unrelated and ambiguous conditions was also calculated by identifying the point at which each difference wave reached 50% of its peak amplitude. This data was entered into a repeated measures ANOVA with group, condition, hemisphere and region as independent variables. No main effects of group or condition and no interaction effects involving group or condition were evident, indicating a similar N400 onset latency for the unrelated and the ambiguous condition difference waves in both groups.

## Discussion

This research investigated lexical ambiguity resolution during sentence processing in people with PD. It was predicted that PD patients would exhibit difficulties selecting the contextually appropriate meaning of the sentence final ambiguous words, as evidenced by lower accuracy for the ambiguous relative to the unrelated condition, and a smaller or absent N400 for the ambiguous condition relative to the unrelated condition. The results only partially supported these hypotheses.

Beginning with the behavioural results, the control group demonstrated slower RTs and lower accuracy for the ambiguous relative to the unrelated condition. These findings suggest that even for the healthy older adults, the contextually inappropriate meaning of the ambiguous word was at least partially active. The accuracy results contrast with those of the healthy older adults in Faust et al. [26], who displayed no significant difference in accuracy between the ambiguous and unrelated condition at a long ISI. The different findings in the present study may relate to the fact that Faust et al. used a 1000ms ISI, providing more time for ambiguity resolution to occur than the 850ms ISI used in the present study.

Similar to controls, the PD group demonstrated an interference effect for both RT and accuracy, however the interference effect for accuracy was significantly larger in PD than controls. These findings suggest that although both groups may have failed to fully suppress the contextually inappropriate meaning of the ambiguous word, such difficulties were manifest to a greater extent in PD. This generally accords with other indications from behavioural research that PD patients experience difficulty using lexical [40,41], sentence [8] as well as discourse [9] information to selectively activate the contextually appropriate meaning of ambiguous words. Thus, the present findings add to existing evidence that the attentional engagement of the semantic network on the basis of context is impaired in PD, potentially due to a deficit in semantic inhibition processes.

Surprisingly, the ERP results were not consistent with the behavioural findings. A similar N400 for the unrelated and ambiguous conditions was evident in both groups, suggesting that the contextually inappropriate meaning of the sentence had been suppressed. Although this finding is consistent with previous ERP studies in healthy adults [23,24], it is inconsistent with the interference effects evident from the behavioural results and also fails to capture the increased accuracy interference effect evident for the PD group. More specifically, based on the difficulties with ambiguity resolution evident from the behavioural data, a reduced N400 effect would be expected for the ambiguous condition, particularly for the PD group. The fact that the same N400 pattern was obtained when errors were included in the ERP analysis further underscores the disparity between the ERP and behavioural findings.

The disparity raises questions about the sensitivity of the different measures to ambiguity resolution difficulties. In an ERP study of how typicality and age of acquisition impact semantic processing, Raling et al. [42] found that although typicality influenced accuracy, reaction time and N400 amplitude, age of acquisition only influenced reaction time. As one potential explanation for why age of acquisition had no impact on the N400, Raling et al. suggested that the N400 was providing an index of early semantic access, whereas age of acquisition effects may originate at later stages of semantic processing or decision making processes. In a similar manner, the interference effects for RT and accuracy in the present study may arise from aspects of processing that are not sufficiently indexed by the N400. Worthy of note, although numerous N400 studies of contextual ambiguity resolution have been conducted [20,22,24,43], the paradigms have not been designed to assess behavioural data in the same manner as the present study. Accordingly, future research should consider including the analysis of both behavioural and ERP data in order to better delineate the nature of ambiguity resolution, and to disentangle the contributions made by behavioural and neurophysiological data to our understanding of this issue.

The ERP results for the PD group warrant further attention. The presence of an N400 for the unrelated condition in PD suggests that semantic processing was generally intact for this sample of patients, and is consistent with previous findings of an intact N400 during sentence processing in PD [18]. The latency of the N400 was also similar for both PD and controls, which contrasts with Angwin et al.’s [17] findings of a delayed N400 in this population. Methodological differences may explain the disparate results, as Angwin et al. used a word pair semantic judgement paradigm that fails to provide the same level of contextual constraint as that used in the present study.

It should also be noted that the correlation analyses indicated that the ERP results may be impacted by individual variability in executive function. Specifically, the analyses indicated that poor Stroop inhibition in the PD group was associated with a smaller N400 magnitude for both the unrelated and ambiguous conditions. This finding appears consistent with suggestions that the N400 provides an index of semantic inhibition [44,45] and adds to existing evidence that executive functions may impact other aspects of language processing impairment in PD, including verb production [27], sentence processing [5,46] and object semantics [46].

In a divided visual field task of semantic processing, Meyer and Federmeier [47] analysed N400 effects and found that healthy older adults with better inhibition were more likely to show a pattern of bilateral activation for dominant meanings. This pattern of bilateral activation was similar to the pattern they previously observed in younger adults [48], suggesting that the older adults with better inhibition retained a pattern of activation similar to their younger counterparts. In a similar manner, the present results could potentially indicate that PD patients with better inhibition are more capable of approximating the lexical-semantic processing skills of healthy controls than patients with lower inhibition. Hence, deficits to ambiguity resolution in PD may be more prominent for patients with reduced inhibition.

The present study emphasises the need to consider individual cognitive profiles when examining language processing impairments in PD, particularly given the heterogeneity of cognitive decline associated with the disease [49]. In addition to lexical ambiguity resolution, inhibitory deficits would also be expected to impact semantic processing more generally. For instance, findings that semantic priming is more prone to disruption in PD [2], including in patients tested off relative to on levodopa medication [50], have been attributed to the potential influence of semantic inhibition deficits in this population. Indeed, the fact that the correlation with Stroop inhibition is evident for both the unrelated and ambiguous conditions highlights the impact of inhibition on semantic processing more broadly.

Of interest, however, is that the same correlation was not evident in the control group despite the fact that both groups performed similarly on the Stroop task. One potential explanation for this finding is that changes to the N400 in PD may be impacted by multi-faceted changes to cognition, of which inhibition is only one component. Further research is needed to identify other cognitive functions that contribute to an altered N400 in this population.

A limitation of the current study is that only one ISI was used, preventing the investigation of any broader changes to the time course of ambiguity resolution. Given behavioural findings of delayed semantic activation in some patients with PD [2,3,51,52], future research should consider the inclusion of multiple ISIs in order to track the time course of ambiguity resolution. Other avenues for future research could also incorporate an analysis of how the strength of sentence context as well as meaning dominance impacts meaning selection in PD. Kotchoubey and El-Khoury [43] demonstrated that meaning activation for a sentence final ambiguous word can be constrained, almost immediately, to even a subordinate meaning when presented within a strong sentence context. Manipulating similar variables in future investigations in PD will assist with further defining the nature of any changes to contextual ambiguity resolution in PD and the potential role of striatal circuitry in this process. Given findings that laterality of symptoms may impact neurophysiological changes during action word processing [53], consideration should also be given to exploring the potential impact of this aspect of PD on the N400.

In conclusion, the behavioural results of the present study revealed ambiguity resolution difficulties for both PD and controls, however these difficulties were manifest to a larger degree in the PD group. Difficulties with ambiguity resolution were not evident in the ERP data, however the results suggested that lexical-semantic impairments may become more evident in PD patients with increased cognitive deficits, particularly those relating to inhibitory processes. Such findings highlight the need to take individual cognitive profiles into account when investigating language processing impairments in this population.
